# There is a long way from current clinical practice in Denmark compared to recent published English guideline on management of children with eosinophilic oesophagitis

**DOI:** 10.1186/s12887-023-04483-3

**Published:** 2024-01-08

**Authors:** Kasper Bredal, Line Tegtmeier Frandsen, Jacob Holmen Terkelsen, Martin Hollænder Nielsen, Dorte Melgaard, Anne Lund Krarup

**Affiliations:** 1 Faculty of Clinical Medicine, Aalborg University, Aalborg, Denmark; 2 Center for Clinical Research, North Denmark Regional Hospital, Hjørring, Denmark; 3 Department of Gastroenterology and Hepatology, Aalborg University Hospital, Aalborg, Denmark; 4 Department of Emergency Medicine and Trauma Center, Aalborg University Hospital, Hobrovej 18-22, Aalborg, DK-9000 Denmark

**Keywords:** Eosinophilic oesophagitis, Eosinophilia, Children, Guidelines, Clinical practice, Complications, PPI, Remission, Treatment

## Abstract

**Background:**

A low incidence of eosinophilic esophagitis (EoE) in children in the North Denmark Region (NDR) were measured in 2007–2017. Few of the children diagnosed before 2017 were treated to remission suggesting a lack of awareness. While there currently are no guidelines for treating EoE in Denmark, a new English guideline was published in 2022 renewing focus on the disease.

**Objective:**

The aim of this study was to measure the difference of current Danish clinical practice for treatment and follow-up of EoE children in the NDR with the new English guideline from the British Society of Gastroenterology (BSG) and the British Society of Pediatric Gastroenterology, Hepatology and Nutrition (BSPGHAN).

**Methods:**

This retrospective, register-based DanEoE cohort study included 31 children diagnosed with EoE between 2007 and 2021 in NDR. Medical records were reviewed and information about treatment and follow-up were collected.

**Results:**

In 32% of the children with EoE in the NDR, first-line treatment corresponded with the new English guideline. One in 6 children were never started on any treatment even though treatment always is recommended. Histologic evaluation within 12 weeks as recommended was performed in 13% of the children.

**Conclusions:**

In Denmark focus on improving EoE treatment and follow-up for children is needed, as there is a significant difference between current clinical practice and the recommendations in the new English guideline.

**Supplementary Information:**

The online version contains supplementary material available at 10.1186/s12887-023-04483-3.

## Introduction

Eosinophilic oesophagitis (EoE) is a chronic, immune-mediated disease of the oesophagus characterized by oesophageal dysfunction, and inflammation with ≥15 eosinophils per high-power field (hpf) in the oesophageal epithelium [1]. EoE affects both adults and children [1]. The prevalence among children in Europe is 41/100,000 with an increasing incidence [2]. More adults than children are diagnosed with EoE [2], which may partly be explained by the complex symptomatology of the disease in children, resulting in lack of detection [3]. Infants, toddlers or young children may present with abdominal pain, heartburn, vomiting, food avoidance, and failure to thrive [3]. Typical symptoms for adolescents are dysphagia and food bolus obstruction [3]. If untreated EoE can lead to the development of oesophageal strictures, psychiatric comorbidity, and low quality of life [1, 4]. EoE is often easy to treat with Proton Pump Inhibitors (PPIs) or topical corticosteroids [5, 6]. Elimination diets are also a possibility but require more endoscopies and may have social side effects along with poor compliance [7]. Due to the heterogeneity of EoE several guidelines have been published to help clinicians to navigate and secure evidence-based treatment [8–14]. In Denmark, a national guideline for treatment of EoE in children does not exist and the tradition has been to use the ESPHGAN guideline from 2014 [9]. For the entire country a very low incidence has been found using the medical registries, probably mostly explained by a lack of detection [15]. In the North Denmark Region (NDR) all EoE children’s medical records were reviewed in a quality project for children diagnosed in 2007–2017 [16]. The project showed a diagnostic delay of more than 4 years, which is more than twice as long as compared to studies of other European countries [17]. Furthermore, these children were rarely treated and followed up according to the ESPHGAN guideline from 2014 [9, 16]. Since then, several efforts have been set in motion to raise awareness of EoE treatment in adults. It is thought to have affected the pediatric population too. In 2022 a new guideline from the British Society of Gastroenterology (BSG) and the British Society of Pediatric Gastroenterology, Hepatology and Nutrition (BSPGHAN) was published and bring EoE into focus again [9, 10]. The aim of this study was to measure the difference of the current clinical practice for treatment and follow-up of EoE children in the NDR with the BSG and BSPGHAN 2022 guideline.

## Methods

### Study population

This is a retrospective, population- and registry-based study of children in the DanEoE2 cohort. The DanEoE cohort has previously been described in detail [18]. Briefly, all citizens having an EoE diagnosis and living in NDR are included in the cohort by use of the pathology registry [19]. The pathology registry in Denmark is among the best in the world [20, 21]. The inclusion criteria for the cohort were at least one oesophageal biopsy with 15 or more eosinophils in one hpf between 2007 and 2017 (DanEoE) and 2018–2021 (DanEoE2). Exclusion criteria were living outside the NDR, and for this study age ≥ 18 years at diagnosis or not fulfilling the AGREE criteria for EoE [22]. In the pediatric part of the cohort all medical records, endoscopies and histology reports were reviewed and entered in the database by a medical student and discussed with an experienced gastroenterologist (ALK) for validation. We compared the BSG and BSPGHAN 2022 guideline with the clinical practice in the DanEoE children from 2007 to 2021 to establish differences. The NDR is a geographically well-defined area with approximately 600,000 citizens, of which 120,000 are children. The composition of the citizens resembles the other four regions in Denmark ensuring a high external generalizability [15]. All Danish citizens have free access to the health care system. In Denmark, all individuals are assigned a unique security number that links all medical record including laboratory investigations, pathology, microbiology and radiology results [20, 21].

### The BSG and BSPGHAN 2022 guideline and recommendations

The BSG and BSPGHAN 2022 guideline is evidence-based recommendations for the diagnostics and management of EoE in adults and children [10]. There are a total of 57 statements. The statements focusing on treatment and follow-up in children are presented in Supplemental Table 1. Briefly, first-line treatment may include high-dose PPIs, specific diets, or topical corticosteroids. The BSG and BSPGHAN 2022 guideline recommends omeprazole 20 mg twice a day or equivalent. In this study omeprazole 20 mg twice a day is considered equivalent to pantoprazole 40 mg twice a day, lansoprazole 30 mg twice a day, esomeprazole 20 mg twice a day, or rabeprazole 20 mg twice a day. First-line treatment was defined as the first initiated therapy after eosinophilia was shown in a biopsy from the oesophagus. If the treatment failed, and a new treatment was initiated, it was considered a second-line treatment, whereas a dose change or change of diet was not.

**Fig. 1:**
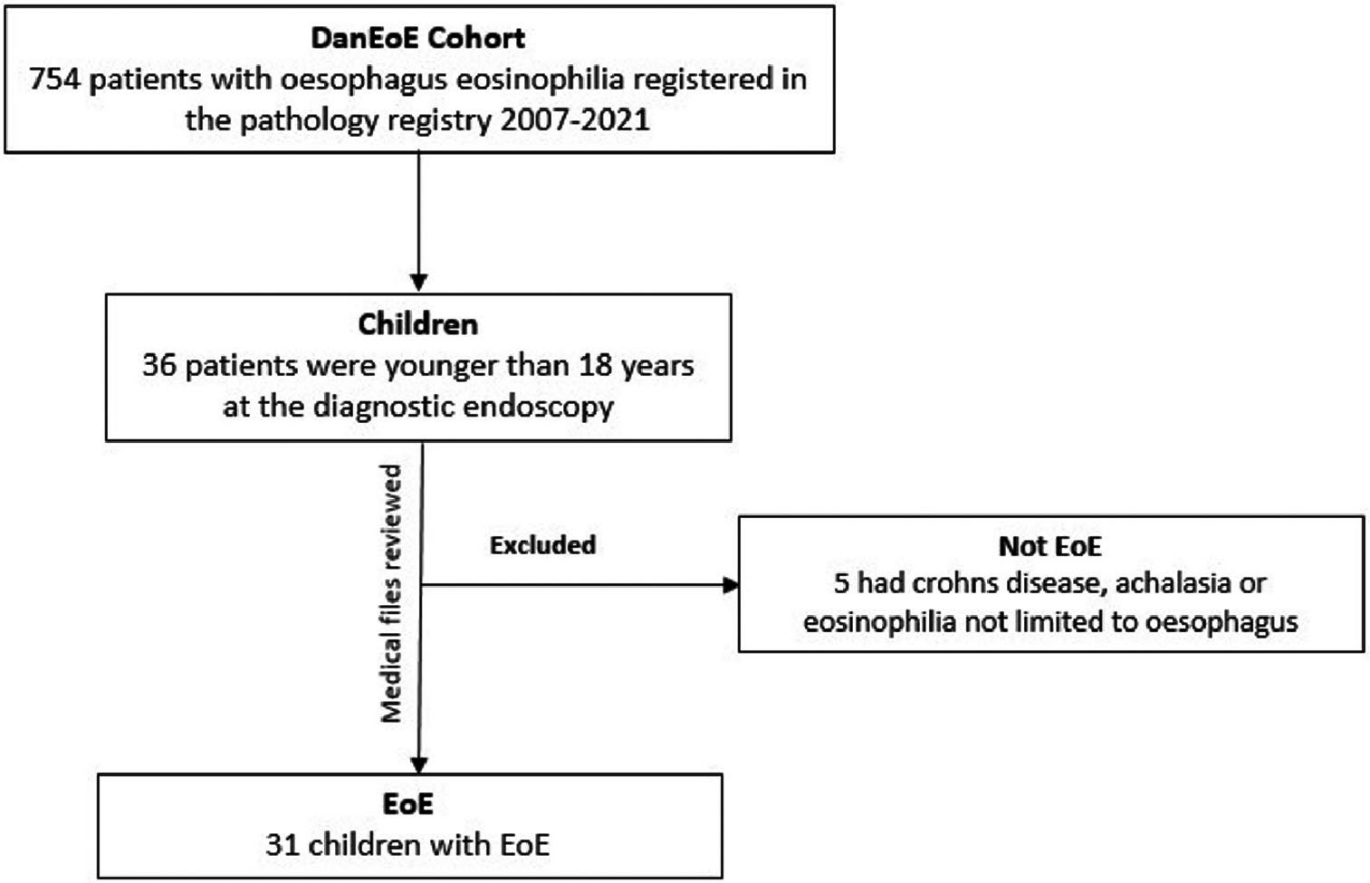
Flowchart for the inclusion and exclusion process

### Statistics

Descriptive staticitcs were given as median and range (25–75 percentile [IQR]) or mean (stardarddeviation [SD]) for continuous varible as appropriate. Groups were compared with a Wilcoxon Rank Sum test. For categorical variables, counts and percentages were displayed. Incidence of our study population was calculated on the basis of data from the governmental institution Statistics Denmark (dst.dk). *P* < 0.05 were considered statistically significant. The SAS 9.4 (SAS Institute Inc., Cary, Nc., USA) was used to perform the data management and statistics.

## Results

Between 2007 and 2021 a total of 31 children was confirmed with EoE in the NDR (Fig. 1). Of the 31 patients diagnosed with EoE, 18 of the patients were diagnosed between 2007 and 2017 and 13 of the patients between 2018 and 2021. Data on the 18 patients diagnosed between 2007 and 2017 are previously published (DanEoE cohort) [16]. Differences compared to children diagnosed after 2017 (DanEoE2 cohort) will be presented in this study.

### The DanEoE cohort and DanEoE2 cohort differences

Of the 13 children diagnosed between 2018 and 2021 (DanEoE2 cohort) 77% were males (10/13) and 23% were females (3/13). The median age at debut was 13 (11;15) years, this had not changed compared to 2007–2017 (*p* = 1.0). The median diagnostic delay was 123 weeks (60;393), which was numerically 19 weeks longer compared to 2007-17, but not statistically significant (*p* = 0.6). The incidence for EoE in children in 2018–2021 was 2.9/100,000 which was a numerical increase of 2.04/100,000 compared to 2007–2017.

### Clinical practice in the NDR from 2007 to 2021 compared to the new guideline: First-line treatment and follow-up

#### The BSG and BSPGHAN 2022 guideline recommends first-line treatment as either high-dose PPIs BID, topical corticosteroid, or elimination diet with a step-up approach starting with 2 food elimination diet

**Fig. 2:**
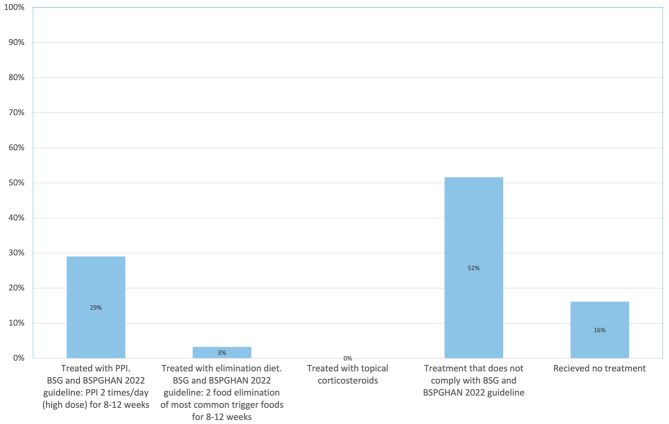
First-line treatment of EoE in children. The figure shows the percentage of cases with current clinical practice in the North Denmark Region in 2007–2021 that were in line with the new BSG and BSPGHAN guideline from 2022

First-line treatment in consistent with the new guideline was initiated in 32% (10/31) of all patients and 31% (4/13) of the patients after 2017. One patient was treated with 2 food elimination diet, and the rest with high-dose PPIs BID (Fig. 2). Details are presented in Supplemental Table 2. Other treatment options not included in the new 2022 guideline was initiated in 52% (16/31) of the children (Fig. 2). This included different types of diets, low-dose PPIs, or a combination of these. Five patients (16%) were never treated (Fig. 2).

#### The BSG and BSPGHAN 2022 guideline recommends systematic follow-up with both symptomatic and histologic evaluation within 12 weeks

**Fig. 3:**
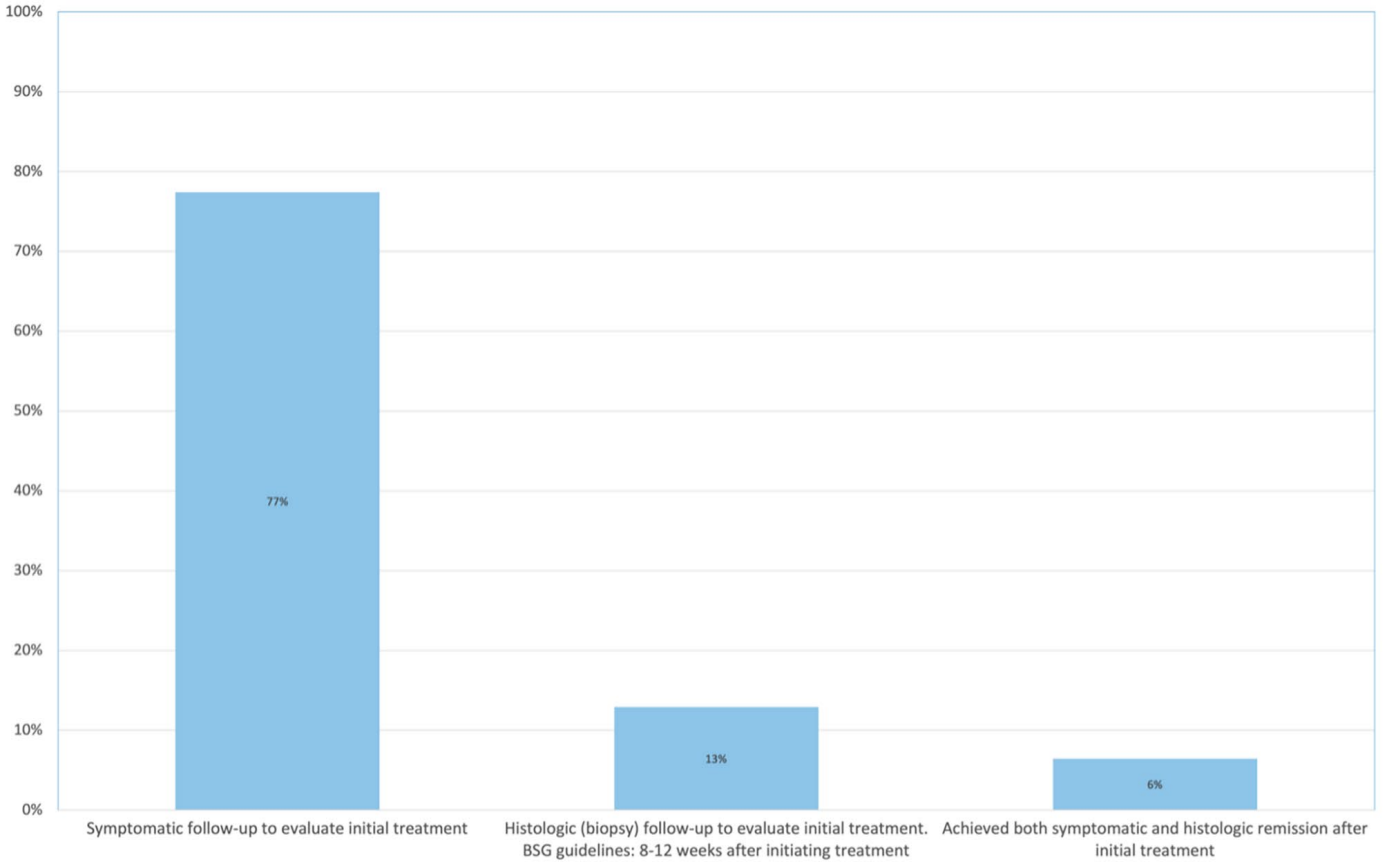
Percentage of clinical practice in North Denmark Region from 2007–2021 in line with follow-up after initial treatment recommended by the BSG and BSPGHAN.

In the NDR symptomatic follow-up was completed in 77% (24/31) of children (Figs. 3) and 85% (11/13) of the children after 2017. Histologic follow-up within 12 weeks was completed in 13% (4/31) of the children (Figs. 3) and 15% (2/13) of the children after 2017. Combined symptomatic and histologic remission after first-line treatment was achieved in 6% (2/31) within 12 weeks (Fig. 3).

### Clinical practice in the NDR from 2007 to 2017 compared to the new guideline: Second-line treatment, follow-up, and maintenance therapy

#### The BSG and BSPGHAN 2022 guideline: If the initial treatment fails, it is recommended to start a second-line treatment in all cases

In the NDR second-line treatment consistent with the new guideline was started in 22% (5/23) of the patients without symptomatic and histologic remission after initial treatment. One child corresponding to 4% (1/23) achieved combined symptomatic and histologic remission after second-line treatment.

#### Follow-up recommendations after second-line treatmet are the same as after first-line

Only one child received third-line treatment. The child achieved symptomatic remission, but not histologic remission.

In total 10% (3/31) of the patients achieving combined symptomatic and histologic remission when efficacy of all treatments started were counted.

If the time aspect was not considered, a total of 42% (13/31) children were rebiopsied at some point. Of the patients who were rebiopsied, 30% (4/13) were rebiopsied after being transferred to an adult gastroenterologist at age 18.

#### The BSG and BSPGHAN 2022 guideline recommends a maintenance treatment to prevent relapse

Maintenance treatment after ensured symptomatic and histologic remission was prescribed in 3% (1/31) of all the patients.

## Discussion

This retrospective, register-based study of children with EoE in the NDR from 2007 to 2021 showed that current clinical practice is considerably different compared to the new English guideline from BSG and BSPGHAN. First-line treatment recommendations corresponded to 32% of treatments administrated in the children in the NDR. One in 6 children did not start on any treatment. Histologic evaluation within 12 weeks as recommended was performed in 13% of the children.

### Study population and incidence

A rising incidence from 0.86 to 2.9/100,000 after 2017 was observed, indicating an increased recognition of the disease. Increasing of the EoE incidence is a national phenomenon documented by the Danish registry study by Allin et al. [15]. However, the increase in children is much lower than seen in adults and in general lower than the European average and globally [2, 15]. The median age in the current study was 13 years and unchanged between the two cohorts. In a retrospective, multicenter study of 410 children diagnosed between 1999 and 2016 from 26 European pediatric gastroenterology centers by Hoofien et al. the median age was reported to be 9.1 years [23]. A cross-sectional study from Spain with 148 prospectively recruited children with EoE diagnosed between 2014 and 2016 reported a median age of 10.43 years, but this study only recruited children under the age of 15 years [24]. The higher median age observed in our study indicates that the awareness of EoE in younger children needs improvement in Denmark. A considerable reason could be an insufficient focus of the disease in Medical Education activities. Particularly when educating Pediatricians and General Practitioners. Also, the unspecific symptomatology seen in younger children make them more difficult to diagnose. EoE is especially seen among atopic children [23], and the disease should therefore especially be considered in patients with atopy or other allergic diseases.

### Differences between the ESPHGAN 2014 guideline used in Denmark up to 2022, and the recent published BSG and BSPGHAN 2022 guideline

Since pediatricians in Denmark never have had a national guideline for EoE, the ESPHGAN guideline from 2014 have been recommended for clinical use. When comparing the 2014 and the 2022 guidelines, there are notable differences. The differences concerning treatment are (1) PPIs are no longer a trial to exclude gastro-oesophageal reflux disease (GORD), but considered as a treatment option, and (2) step-up elimination diet starting with a two-food elimination has replaced targeted elimination diet of specific food triggers based on allergy testing [9, 10]. Treatment lengths and follow-up regiments are unchanged in the two guidelines [9, 10].

### Management and treatment plan

Most of the children diagnosed with EoE started on treatment and almost all of them started PPI treatment. In most cases the dose was too low or was only taken once per day, suggesting that treatment reflected other possible diagnosis than EoE e.g., reflux among the pediatricians. Low-dose PPIs were often initiated in the children due to reflux symptoms, and the treating physician might have forgotten to increase the dose later, when the EoE diagnose was confirmed. The recommendations to divide the treatment in two doses per day is based on a meta-analysis from Lucendo et al. [25] that describes a non-significant trend towards increased efficacy for two times per day dosing compared with a onetime per day dose. Even though two doses per day are preferable and recommended, it is likely that some patients might have better compliance with one high dose PPI per day. The second most common treatment was diet treatment, but rarely in accordance with previous or current EoE guidelines. Histologic evaluations were rarely performed. General anesthesia and endoscopies might worry parents and clinicians in Denmark, but it is found to be safe [26, 27]. Symptoms do not correlate well with oesophageal inflammation and should not be used as the sole measure of disease activity [28]. Therefore, repeat biopsies are important to assed disease activity. Asymptomatic patients may still have inflammation, and untreated patients are believed to have an increased risk of fibrotic disease with stricturing in the future [29]. When comparing the period before and after 2017 regarding first line treatment and follow up within 12 weeks, there where almost no improvement. This add to the argument that medical activities have been lacking up till 2021 and more education is needed. Second-line treatment was started in one of five cases where this was relevant. Mostly the second-line treatment was treatment with topical corticosteroids. The impression from reviwing all the medical records is that Danish clinicians are reluctant to prescribe topical corticosteroids to children. This is unfortunate as topical corticosteroids are very effective with few and mild side effects e.g., oral candidiasis but not growth retardation, adrenal insufficiency, diabetes or osteoporosis [10, 23, 30]. Treatment with topical corticosteroids was only started if parents were recommended this by a specialist, indicating that EoE treatment would benefit from standardizing treatment in Denmark using a nationally recognized guideline.

### Strength and limitations

The findings from the NDR is expected to be comparable to the rest of the country as the Danish regions have a very similar demographic [31]. The population-based cohort based on the pathology registries, followed by review of the medical records, and diagnosis based on the AGREE consensus is thought to ensure validity of data. However, it is a small study emphasizing the detection problems we have with EoE children. This has been shown in a national registry study to be a problem in all Danish regions [15]. With the current Danish practice there is a risk that many of the diagnosed EoE children are insufficiently treated. This study suggests that EoE treatment needs national attention.

## Conclusion

Focus on improving treatment and follow-up for children diagnosed with EoE is still needed in Denmark, where significant differences between current clinical practice and the recommendations in the BSG and BSPGHAN 2022 guideline are observed.

### Electronic supplementary material

Below is the link to the electronic supplementary material.

Supplementary Material 1

Supplementary Material 2

## Data Availability

The datasets used and/or analysed during the current study are available from the corresponding author on reasonable request.

## Abbreviations

EoE: Eosinophilic oesophagitis
NDR: North Denmark Region
BSG: British Society of Gastroenterology
BSPGHAN: British Society of Pediatric Gastroenterology
PPI: Proton Pump Inhibitor
Hpf: High power field
GORD: Gastro-oesophageal reflux disease
