# Supplementary material for: There is a long way from current clinical practice in Denmark compared to recent published English guideline on management of children with eosinophilic oesophagitis
Source: BMC Pediatr. 2024 Jan 8;24:24. doi: 10.1186/s12887-023-04483-3 (PMC10773032; doi:10.1186/s12887-023-04483-3)
Supplement: Supplementary file 1 — Supplementary Material 1 [file 12887_2023_4483_MOESM1_ESM.docx]

Supplemental Table 1: The BSG and BSPGHAN 2022 guideline statements focusing on treatment and follow-up.
The table shows the statement on the left column and level of recommendation on the right.
All recommendations are published in Dhar A. et al. (9).

| BSG and BSPGHAN 2022 guideline | |
| --- | --- |
| **Treatment and management** | **Grade of evidence and level of recommendation** |
| After initiation of therapy (dietary or pharmacological treatment), endoscopy with biopsy while on treatment, is recommended to assess response, as symptoms may not always correlate with histological activity. | GRADE of evidence: Low Level of recommendation: Strong |
| Elimination diets are effective in achieving clinicohistological remission in both adults and paediatric patients with eosinophilic oesophagitis. | GRADE of evidence: Moderate.  Level of recommendation: Strong. |
| A six food elimination diet results in higher histological remission rates than two or four food elimination diets but is associated with lower compliance and an increased number of endoscopies. | GRADE of evidence: Low.  Level of recommendation: Strong. |
| When undertaking a dietary restriction, support from a dietitian throughout the elimination and reintroduction process is strongly recommended | GRADE of evidence: Low.  Level of recommendation: Strong. |
| Allergy testing to foods (eg, skin prick, specific IgE and patch testing) is not recommended for choosing the type of dietary restriction therapy for eosinophilic oesophagitis. | GRADE of evidence: Low  Level of recommendation: Strong. |
| Exclusive elemental diets have a limited role in eosinophilic  oesophagitis, with high efficacy but low compliance rates and should be reserved for patients refractory to other treatments. | GRADE of evidence: Low  Level of recommendation: Strong. |
| Proton pump inhibitor therapy is effective in inducing histological and clinical remission in patients with eosinophilic oesophagitis. | GRADE of evidence: Moderate  Level of recommendation: Strong. |
| Proton pump inhibitor therapy should be given two times per day for at least 8–12 weeks prior to assessment of histological response, while on treatment. | GRADE of evidence: Low.  Level of recommendation: Strong. |
| In patients who achieve histological response, proton pump  inhibitor therapy appears effective in maintaining remission. | GRADE of evidence: Low.  Level of recommendation: Strong. |
| Topical steroids are effective for inducing histological and clinical remission in eosinophilic oesophagitis. | GRADE of evidence: High.  Level of recommendation: Strong. |
| Clinical and histological relapse is high after withdrawal of topical steroid treatment, and following clinical review, maintenance treatment should be recommended. | GRADE of evidence: Moderate.  Level of recommendation: Strong. |
| Systemic steroids are not recommended in eosinophilic  oesophagitis. | GRADE of evidence: High.  Level of recommendation: Strong. |
| Immunomodulators (eg, azathioprine, 6-mercaptopurine) are not recommended in the management of eosinophilic oesophagitis. | GRADE of evidence: Low.  Level of recommendation: Weak. |
| Monoclonal antibody therapies, such as anti-tumour necrosis  factor (TNF) and anti-integrin therapies, that are typically used for inflammatory bowel disease are not recommended in the management of eosinophilic oesophagitis. | GRADE of evidence: Low.  Level of recommendation: Weak. |
| Novel biologics used in other allergic conditions (such as  dupilumab, cendakimab and benralizumab) have shown promise in the treatment of eosinophilic oesophagitis. | GRADE of evidence: Low.  Level of recommendation: Weak. |
| Sodium cromoglycate, montelukast and antihistamines are not recommended in the management of eosinophilic oesophagitis but may have a role in concomitant atopic disease. | GRADE of evidence: Moderate.  Level of recommendation: Strong. |
| If symptoms recur while on treatment, we recommend  repeating an endoscopy for assessment and to obtain further histology. | GRADE of evidence: Low.  Level of recommendation: Strong. |
| Patients with eosinophilic oesophagitis refractory to treatment and/or with significant concomitant atopic disease should be jointly managed by a gastroenterologist and specialist allergist to optimise treatment. | GRADE of evidence: Very low. Level of recommendation: Weak. |
