# Supplementary material for: There is a long way from current clinical practice in Denmark compared to recent published English guideline on management of children with eosinophilic oesophagitis
Source: BMC Pediatr. 2024 Jan 8;24:24. doi: 10.1186/s12887-023-04483-3 (PMC10773032; doi:10.1186/s12887-023-04483-3)

**Supplemental Table 2:** Overview of treatments. The table shows an overview of the different treatments. High dose Proton Pump Inhibitor (PPI) treatment is defined as 2 mg/kg omeprazole, reaching a maximum dose equivalent to 40 mg for adults, which is considered comparable to 80 mg pantoprazole, 60 mg lansoprazole, and 40 mg esomeprazole. Low dose treatment is defined as

1 mg/kg, totaling 20 mg omeprazole or equivalent. The minimum weight of children treated with PPI was 27.3 kg, and no children under the age of 7 received PPI treatment. Recommended treatments are marked dark grey. Each row represents a child diagnosed with EoE. The columns represent the number of treatment and whether or not the child received remission.


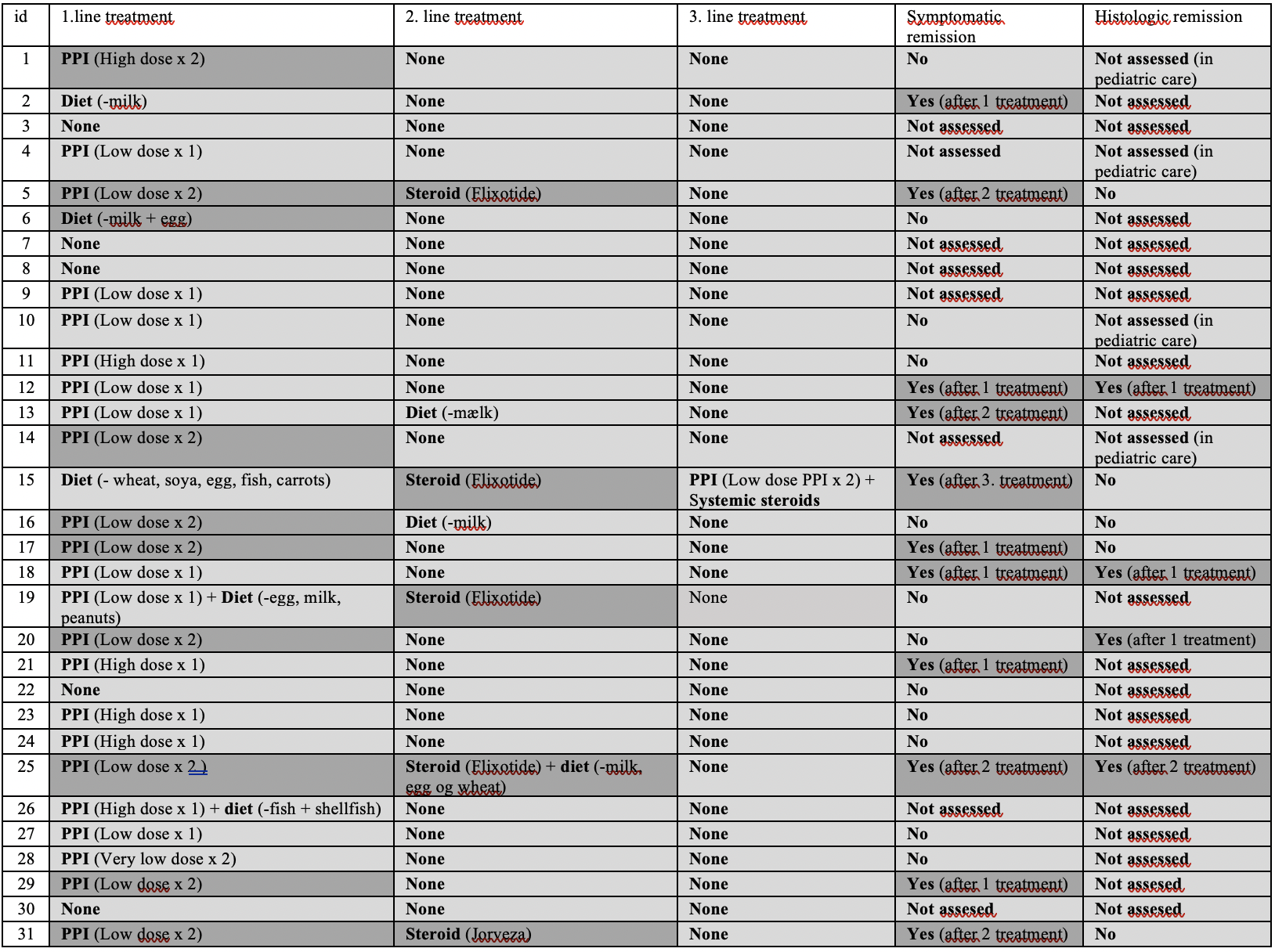

Supplement: Supplementary file 2 — Supplementary Material 2 [file 12887_2023_4483_MOESM2_ESM.docx]
